# Network fingerprint: a knowledge-based characterization of biomedical networks

**DOI:** 10.1038/srep13286

**Published:** 2015-08-26

**Authors:** Xiuliang Cui, Haochen He, Fuchu He, Shengqi Wang, Fei Li, Xiaochen Bo

**Affiliations:** 1 Beijing Institute of Radiation Medicine, 27 Taiping Road, Beijing 100850, China; 2 International Cooperation Laboratory on Signal Transduction, Eastern Hepatobiliary Surgery Institute, Second Military Medical University, Shanghai 200433, China

## Abstract

It can be difficult for biomedical researchers to understand complex molecular networks due to their unfamiliarity with the mathematical concepts employed. To represent molecular networks with clear meanings and familiar forms for biomedical researchers, we introduce a knowledge-based computational framework to decipher biomedical networks by making systematic comparisons to well-studied “basic networks”. A biomedical network is characterized as a spectrum-like vector called “network fingerprint”, which contains similarities to basic networks. This knowledge-based multidimensional characterization provides a more intuitive way to decipher molecular networks, especially for large-scale network comparisons and clustering analyses. As an example, we extracted network fingerprints of 44 disease networks in the Kyoto Encyclopedia of Genes and Genomes (KEGG) database. The comparisons among the network fingerprints of disease networks revealed informative disease-disease and disease-signaling pathway associations, illustrating that the network fingerprinting framework will lead to new approaches for better understanding of biomedical networks.

High-throughput experimental technologies enabled biological studies be performed at the network scale in recent years. Various molecular networks (protein-protein interaction, metabolic, signaling and transcriptional regulatory networks) have become basic objects in biomedical studies^1^. Powerful computational tools are therefore needed to help researchers gain insight into the meaning of biological networks. Several approaches have been proposed to decipher these complex objects, such as identifying topological important nodes, finding structural motifs^2^, and detecting communities^3^,^4^. However, these approaches may involve too many new mathematical concepts, and cannot provide a familiar “language” for biomedical researchers that clearly indicate biological functions or diseases. Unlike researches on other kinds of complex networks, in biomedical studies, a number of basic networks with clear functions, referred to as “pathways” or “modules”, provide a good basis for knowledge-based exploration of biomedical networks. For example, the well-known KEGG database now contains a large number of manually-created pathways representing current knowledge of molecular interactions and reaction networks for several processes, including metabolism, genetic information processing, and cellular processes^5^. This situation inspired us to adopt the comparison and decomposition strategies for molecular network understanding.

In this study, we introduce a framework to characterize a biomedical network using a series of its similarities (both structural and functional) to a set of basic networks, which we call “network fingerprint”. Given a set of basic networks 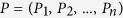, a biomedical network *G* can be characterized by a network fingerprint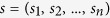, where *s*_*i*_ = *sim* (*G*, *P*_*i*_) is the functional similarity between *G* and *P*_*i*_. It is important to choose proper basic networks set. Here, we use the well-studied KEGG signaling pathways as the set of basic networks. Each of these pathways represents certain cellular process, which is familiar to biomedical researchers. Moreover, the KEGG signaling pathways are relatively independent with each other, and have proper network size. To compute the network fingerprint, we presented an algorithm to measure the functional and structural similarity between *G* and *P*_*i*_ based on the gene ontology (GO) and affinity propagation (AP) clustering algorithm^6^, and the similarity score is normalized by the random simulation procedure (details are shown in the Supplementary files). The network contains a sub-network similar to a certain basic network have a corresponding high score. In fact, there are already several methods to measure the similarity of two networks in computer science. These methods are almost based on topological structure of networks but ignore the network node property. However, the biological function of the proteins is an important factor when compare two biomedical networks. Differently, our method takes both the topological structure and the function of the proteins in the network into consideration. Based on this approach, we provide new insights into the space of disease networks as well as the relationships between diseases and signaling pathways.

## Results

### Network fingerprint of Type 1 diabetes mellitus and Type 2 diabetes mellitus

**Figure 1:**
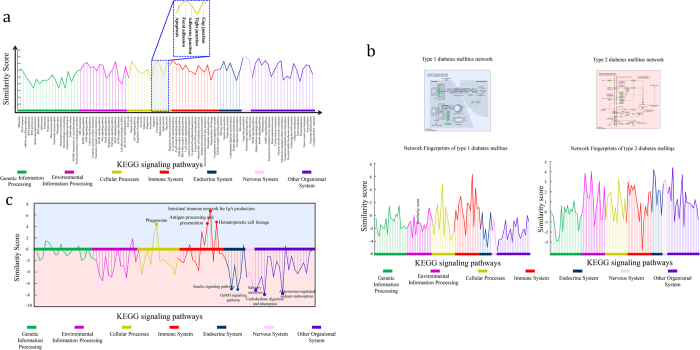
The concept map of network fingerprint (taking T1DM and T2DM as examples). (**A**) The structure of network fingerprint of disease network based on the KEGG signaling pathways. The Different kinds of pathways are represented as different colors. (**B**) Molecular network of T1DM and T2DM in KEGG database and their corresponding network fingerprints. (**C**) The difference between the network fingerprints of T1DM and T2DM.

To demonstrate how network fingerprint is a better approach to characterize biomedical networks, especially for disease networks, we used 93 well-studied KEGG signaling pathways (table S1) as a collection of basic networks. The disease networks were downloaded from the KEGG pathway database (table S2), which had been drawn manually based on current knowledge about these diseases. Based on our network fingerprint extracting algorithm, we obtained the fingerprints of these disease networks including 93 similarity scores compared to signaling pathways in the KEGG database (Fig. 1A).

The network fingerprints have a more familiar form and can give more clear meanings for the biologists. As an example, the network fingerprint of the two basic types of diabetes, Type 1 diabetes mellitus (T1DM) and Type 2 diabetes mellitus (T2DM), are illustrated in Fig. 1B. Although the two diseases are both characterized by hyperglycaemia, as their well-known different pathogenesis, the network fingerprints of T1DM and T2DM show significant differences (Pearson correlation coefficient is −0.05) (Fig. 1C).

T1DM is an autoimmune condition characterized by selective autoimmune destruction of pancreatic b-cells. Autoantigen processing and presentation by HLA molecules may play important role in the development of T1DM^7^, and it was reported that the occurrence of IgA deficiency was significantly more common in T1DM patients^8^. Consistently, our analysis show that T1DM has higher association scores with pathways involved in immune system, such as intestinal immune network for IgA production, hematopoietic cell lineage, antigen processing and presentation pathway. While T2DM has higher association scores with aldosterone-regulated sodium reabsorption pathway, carbohydrate digestion and absorption pathway, salivary secretion, insulin signaling pathway and GnRH signaling pathway. Our results agree with the hypothesis that sex hormones influence risk factors for T2DM. Moreover, marked hyperglycemia and insulin resistance were found after androgen-deprivation therapy for prostate cancer^9^.

### Disease network classification based on network fingerprint

**Figure 2:**
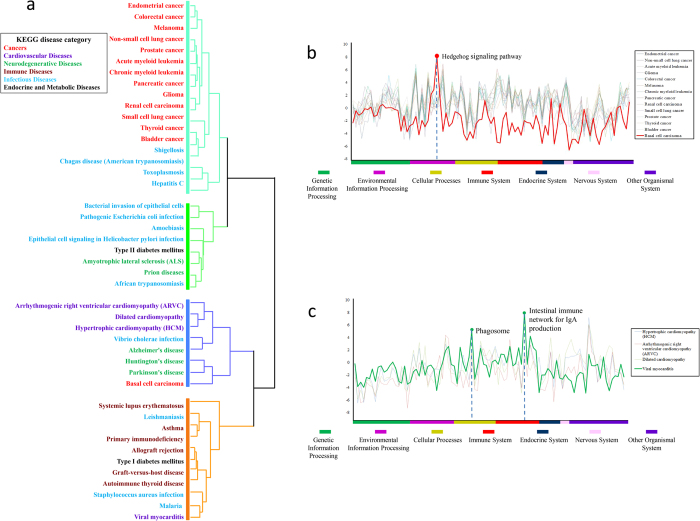
Comparisons of disease network fingerprints. (**A**) The hierarchical clustering of 44 diseases networks based on their fingerprints. The diseases are classified into three groups marked by the color of the dendrogram line. The original KEGG disease classifications are labeled by the color of the font. (**B**) The network fingerprints of 14 cancers. These cancer networks all have similar fingerprints, with the exception of basal cell carcinoma (marked with a red line). (**C**) The network fingerprints of 4 cardiovascular diseases. The fingerprint of the viral myocarditis network, which is significantly different from the others, is marked by a green line.

The network fingerprint also provides a way for systematic comparative analysis on a number of biomedical networks. To explore the space of the disease network, we clustered 44 disease networks in the KEGG pathway database (http://www.genome.jp/kegg/pathway.html#disease) based on their network fingerprints. We utilized hierarchical clustering, using complete linkage method and euclidean as a distance metric (matlab software), and found that these diseases can be significantly classified into 4 groups (Fig. 2A). The first group includes 13 cancers and 4 infectious diseases. Cancers are significantly enriched in this group (*P* < 1.0 × 10^−4^). The second group includes 5 infectious diseases, 2 neurodegenerative diseases and T2DM, and infectious diseases are significantly enriched in this group (*P* < 0.04). The third group includes 3 neurodegenerative diseases, 3 cardiovascular diseases (CVDs), 1 infectious disease and 1 cancer. Neurodegenerative diseases and CVDs are enriched in this group (*P* < 0.04 and *P* < 0.01 respectively). The fourth group includes 6 immune diseases, 1 CVD, 3 infectious diseases, and T1DM. Immune diseases are enriched in this group (*P* < 1.0 × 10^−4^). Using the Kappa statistic, we evaluated inter-observer agreement between this classification and the manual classification in KEGG. The Kappa index showed a substantial agreement (*Kappa* = 0.70; *P* < 1.0 × 10^−10^). Although the clustering result of diseases based on their network fingerprints is in good agreement with the classification of KEGG, there are some suggestive exceptions.

The network fingerprints of four infectious diseases, caused by shigellosis, American trypanosomiasis, toxoplasmosis and Hepatitis C virus (HCV), are clustered into the cancer enriched group, indicating a close relationship between viral infection and cancer. For HCV, there is strong epidemiologic evidence showing that HCV infection is a leading cause of hepatocellular carcinoma (HCC)^10^,^11^.

We also noticed that the network of prion disease is clustered together with some infectious diseases. For example, there are many hypotheses about composition of infectious prions and the mechanism of their formation in the neurons of infected hosts; none has yet been proven^12^. The similarity between network fingerprints of prion disease and that of other infectious diseases may provide clues for explore the infectious of prion.

The network fingerprints of two typical classes of diseases in KEGG, cancers and CVDs, are shown in Fig. 2B,C respectively. As illustrated in Fig. 2B, basal cell carcinoma (BCC) has a very different network fingerprint from other cancer types (the average pearson correlation coefficient between BCC and other 13 cancers is 0.1436, while the average correlation coefficient among the other 13 cancers is 0.7325). We notice that the network fingerprint of BCC had a specifically high similarity score with Hedgehog signaling pathway (the similarity score is 8.1538, while the average similarity between Hedgehog and other cancers is 1.0213). Studies have shown consistent overexpression of PTCH, which is a negative regulator of the Sonic Hedgehog signaling pathway in BCC, indicating that the development of BCC is associated with altered activity of the members in this pathway^13^,^14^. However, BCC metastatic is rare, indicating pathway having high similarity score with other cancers and low similarity score with BCC, such as VEGF, ErbB and mTOR, may play important role in cancer metastatic^15^,^16^,^17^,^18^.

The clustering of disease network fingerprints also shows that viral myocarditis had different network fingerprint with other 3 CVDs (Fig. 2C) (the average Pearson correlation coefficient between viral myocarditis and other 3 CVDs is 0.3653, while the average correlation coefficient among the other 3 CVDs is 0.7421). Adenoviruses and enteroviruses such as the coxsackieviruses have been implicated as causes of myocarditis^19^. The network fingerprint shows that viral myocarditis has a specific high association with pathways involved in viral entry into the cell such as phagosome and cell adhesion molecules (CAMs) pathways (the similarity scores are 5.0937 and 2.578 respectively, while the average similarity scores between these two pathways and other CVDs are 1.0114 and 1.4101 respectively).

### Relationship between disease and signaling pathways

**Figure 3:**
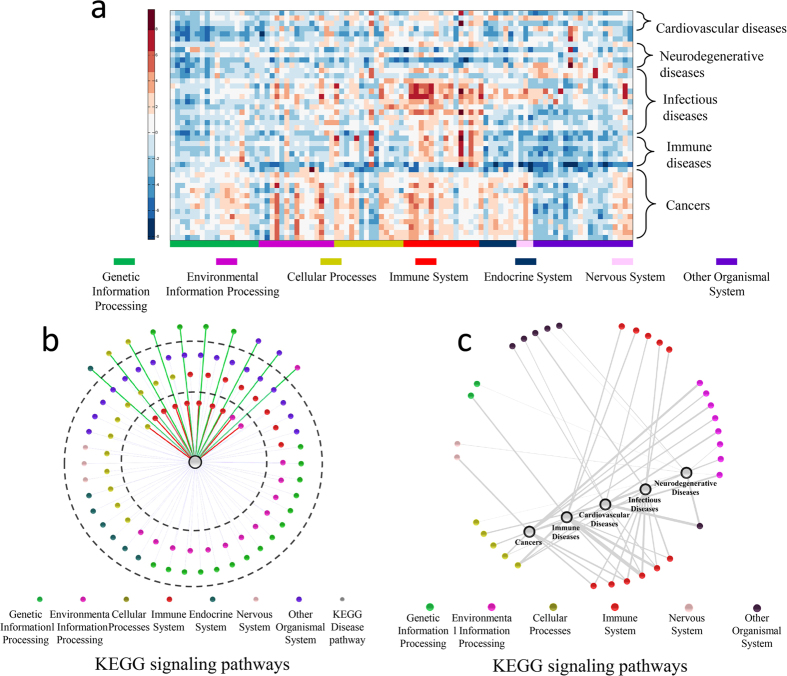
Disease-signaling pathway relationship based on network fingerprint. (**A**) The heat map of 44 disease network fingerprints. The underlying data are based on functional and structural similarities between 44 disease networks and 93 KEGG signaling pathways, and the signaling pathway categories are marked according to the KEGG classification. (**B**) The connection map of universal disease and signaling pathways, in which the nodes for closely related pathways to a universal disease (small circle in the innermost ring) is connected to the disease node (the middle circle) by a red line, and green indicates the nodes for poorly-related pathways to a universal disease (small circles in the outermost ring) connected to a universal disease node (in the middle). The pathway nodes (small) are colored according to manual KEGG disease classification. Color codes are given in the legend. (**C**) The connection map of category specific disease and signaling pathways, in which a disease category node (large blue circles) and a signaling pathway node (small circles with color) are connected to each other by a grey line if the signaling pathway is one of the ten most sensitive pathways. Line thickness is proportional to the similarity score between the disease category node and signaling pathway node. The pathway nodes (small) are colored according to manual KEGG disease classification. Color codes are provided in the legend.

Based on the network fingerprints, we also explored global relationships between the 44 diseases and the 93 signaling pathways. The heat map of the 44 disease network fingerprints (Fig. 3A) illustrate that the same type of diseases may have different patterns of network fingerprints and the same signaling pathway can be activated in different diseases. In particular, we focused on three kinds of signaling pathways: those with close relationships with most diseases, those with poor relationships with most of diseases, and those with close relationships with specific disease types. Here, we use the numerical average of the 44 diseases network fingerprints to represent the fingerprint of “most of disease”.

Figure 3B illustrates the connective map of signaling pathway and disease based on network fingerprint. The top 10 signaling pathways with close relationships to most of the diseases (Table S3) and the top 10 signaling pathways with poor relationships to most of the diseases (Table S4) are located in the inner and outer loops, respectively. 7 of the top 10 signaling pathways with close relationships to most of diseases are involved in the immune system, indicating that the immune system plays a significant role in a large number of human diseases. The other 3 pathways, including ErbB signaling pathway, apoptosis and Jak-STAT signaling pathways, are important for cell proliferation, differentiation and apoptosis. These pathways have been recognized to play key roles in cancer development^20^, neurodegenerative disorders^21^ and pathogen infections^22^. 4 of the top 10 signaling pathways with poor relationships to most of the diseases are highly conserved biological pathways involved in genetic information processing. The other 6 pathways with low correlations to the most of the diseases are involved in the transport system and other organismal system.

The 10 signaling pathways with the closest relationships to the 5 types of disease are shown in Fig. 3C. We focus on the signaling pathways having specific close relationships with certain type of disease, and low relationships with other type of disease. Our network fingerprint results are in agreement with previously published researches. The ErbB, mTOR, TGF-beta and neurotrophin signaling pathways are identified as the cancer-specific pathways according to network fingerprint analysis, which have all believed to play crucial roles in controlling cell growth, proliferation, and survival, and therefore have close relations with cancer. For example, ErbB2 have been realized to be implicated in the development of many cancers^20^, and several promising ErbB-target drugs are in clinical trials^23^,^24^. The mTOR signaling pathway is also believed to be a valuable target for cancer therapy^25^. Five signaling pathways, extracellular matrix (ECM)-receptor interaction, focal adhesion, leukocyte transendothelial migration, vascular smooth muscle contraction and GnRH signaling pathway are identified to be highly correlated with CVDs. ECM is a crucial determinant for adverse cardiac remodeling of cardiomyopathy^26^, and trans-endothelial migration is closely related to myocardial fibrosis^27^. Six pathways, aldosterone-regulated sodium reabsorption, calcium signaling pathway, Long-term potentiation (LTP), proximal tubule bicarbonate reclamation, protein export and peroxisome, have specific and high correlation with neurodegenerative diseases. As a major form of long-lasting synaptic plasticity, LTP is considered to be involved in learning and memory. Cissé *et al*. provided compelling evidence that increasing EphB2 expression can reverse deficits in LTP and memory impairments, which provides a promising therapeutic strategy^28^. The other pathways are also reported to be involved in neurodegenerative diseases progression^29^,^30^,^31^,^32^. The association analysis based on network fingerprints showed that the toll-like receptors (TLRs) signaling, osteoclast differentiation and natural killer (NK) cell mediated cytotoxicity has highly specific correlation with infectious diseases. These pathways are thought to play critical role in innate and adaptive immune responses to viral and bacterial infection^33^,^34^.

## Discussion

Spurred on by the advances in high-throughput experimental technologies, such as microarray, next-generation sequencing and proteomics approaches, data on biological networks are increasing exponentially^1^. Network-scale study has therefore become regular in biomedical studies. In researches on information science, the complex signals, such as audio, images, and video, are always decomposed using base signals for better processing and understanding. Similarly, with the aid of network fingerprinting framework, the biomedical networks can be intuitively deciphered based on the ever-increasing knowledge of protein interactions, signal transduction, transcription regulation and metabolic pathways. Characterizing biological networks as network fingerprints also opens the door for large scale comparisons among physiological and pathogenic networks, which may help identify differences and associations between various biomedical events.

## Methods

The disease networks and signaling pathways were downloaded from the KEGG database^5^ and extracted into graphs employing the R-package KEGGgraph. The function KEGGpathway2Graph of the KEGGgraph was used wih the default parameters (genesOnly = TRUE, expandGenes = TRUE)^35^. More detailed information about the category of these networks and pathways are list in Table S1 and Table S2.

The similarity between two biomedical networks is calculated based on the following intition: grouping the nodes in the merged network into strongly inter-connected communities with high functional similarity score between intra-community nodes in different netwoks The functional similarity was measured based on GO. And we employed affinity propagation (AP)^6^ clustering algorithm to detect the aligned functional modules between the two networks to be compared.

### Network merging

**Figure 4:**
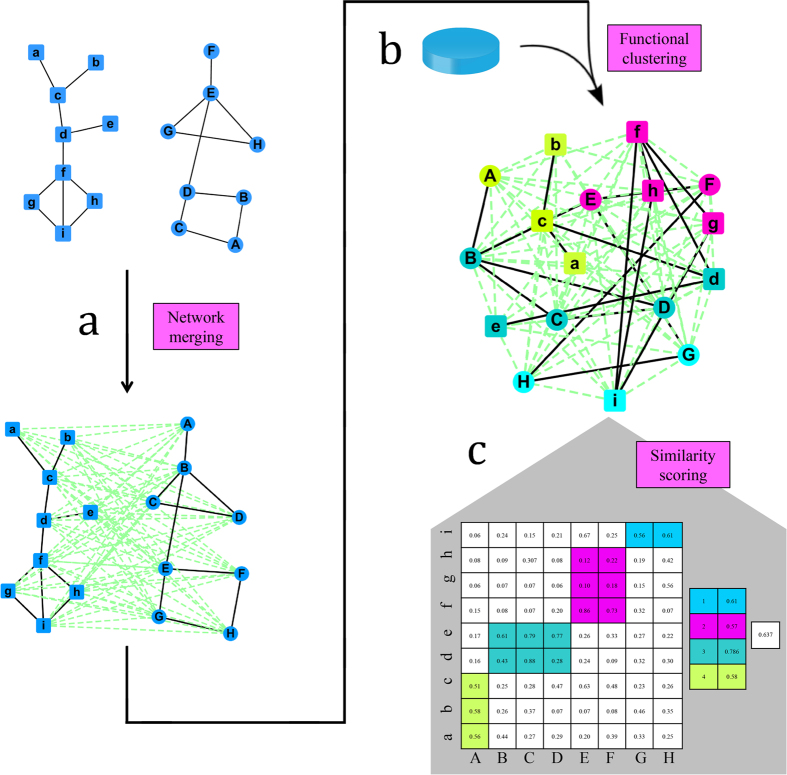
The framework of network fingerprint extraction algorithm. (**A**) Network merging. The networks to be compared, *G*_1_ and *G*_2_, were merged into a virtual integrated fully connected graph G^*^. (**B**) Functional clustering. The edges of G^*^ were weighted by assigning the functional similarity between the interacting nodes based on GO. The AP clustering algorithm was employed to identify the functional modules (represented as different colors) of G^*^. Nodes in a functional module came from *G*_1_ and *G*_2_ (*e.g*., in the red cluster, nodes f, g, and h came from *G*_1_, and nodes E and G came from *G*_2_). (**C**) Similarity scoring. The functional similarity between two networks was calculated based on the clustering results.

The two networks to be compared are first merged into one. Given two networks *G*_1_ = (*V*_1_, *V*_1_) and *G*_2_ = (*V*_2_, *V*_2_), the merged network 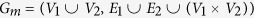 is constructed by connecting each node between the *G*_1_ and *G*_2_ networks (Fig. 4). Two nodes corresponding to the same protein in the merged network are replaced by a single node that inherited all the interactions from the two individual nodes in the subsequent process.

### Annotate network with edge weights

The merged network is annotated with edge weights representing the functional similarity measurement between nodes based on Gene Ontology. Several methods have been presented to measure the semantic similarity, and the evaluation results show that pairwise measures using Resnik’s term similarity^36^ outperform other methods in all studies except family similarity^37^. Here we use Resnik’s similarity in biological process to construct the edge weights. And the weighted adjacency matrix *S*_*m*_ of *G*_*m*_ is defined as follows:


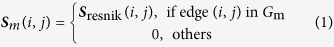


### Group nodes in network

Affinity propagation (AP) algorithm^6^ with default parameters is employed to group nodes of merged network *G*_*m*_ into clusters. The nodes are grouped on the cluster based on nearest neighbor analysis. Suppose the network is divided into N clusters, the subnetwork in each cluster are noted as *C*_*k*_, which are marked by different colors in Fig. 4.

### Similarity scoring

The calculation of similarity score is processed in two steps: local similarity for each cluster and network similarity among cluster.

First, local similarity score *LS*_*k*_ in each cluster *C*_*k*_ of nodes are defined as the mean of the maximum similarity for each pair of nodes between *G*_1_ and *G*_2_ network within cluster *C*_*k*_, but also between different networks. Suppose 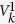 is the set of nodes in both *C*_*k*_ and *G*_1_, same as 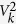, the local similarity score is defined as follows:


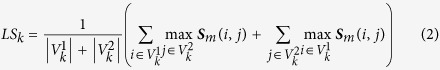


Then, the network similarity score of *G*_1_ and *G*_2_ is defined as the mean similarity of local similarities over all clusters *C*_*k*_ of the merged network *G*_*m*_ as follows:


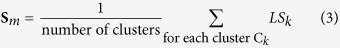


### Standardization

When we compare two networks, the more proteins one network has, the more probable it contains a subnetwork functional similar to the other network. In addition, the network topological structure may also have impact on the similarity score (supplementary Figure S1 and S2), it is necessary to standardize the similarity scores. The standardization procedure is based on the random distribution of the similarity scores. The random networks for estimation have same number of nodes and edges, and have exactly same node degree. The maslov’s method was used to randomize a network while preserving the degree distribution^38^. For a network *G*_2_, a sample size of 1000 randomized networks was served to estimate the background distribution with mean value *E* and standard deviation *X*. And the standardized similarity score between network *G*_1_ and *G*_2_ is defined as follows:


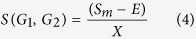


## Additional Information

**How to cite this article**: Cui, X. *et al*. Network fingerprint: a knowledge-based characterization of biomedical networks. *Sci. Rep*. **5**, 13286; doi: 10.1038/srep13286 (2015).

## Supplementary Material

Supplementary Information
