# Supplementary material for: Network fingerprint: a knowledge-based characterization of biomedical networks
Source: Sci Rep. 2015 Aug 26;5:13286. doi: 10.1038/srep13286 (PMC4549786; doi:10.1038/srep13286)
Supplement: Supplementary Information [file srep13286-s1.doc]

Supplementary information

**Network fingerprint: a knowledge-based characterization of biomedical networks**

Xiuliang Cui^1,2^†, Haochen He^1^†, Fuchu He^1^, Shengqi Wang^1^*, Fei Li^1^*, Xiaochen Bo^1^*

1 Beijing Institute of Radiation Medicine, 27 Taiping Road, Beijing 100850, China.

2 International Cooperation Laboratory on Signal Transduction, Eastern Hepatobiliary Surgery Institute, Second Military Medical University, Shanghai, P.R. China.

*To whom correspondence should be addressed. Correspondence and requests for materials should be addressed to: X.-C.B. ([boxc@bmi.ac.cn](mailto:boxc@bmi.ac.cn)) or F.L. ([pittacus@gmail.com](mailto:pittacus@gmail.com)) or S.-Q.W. ([sqwang@bmi.ac.cn](mailto:sqwang@bmi.ac.cn))

†These authors contributed equally to this work.

Supplementary Table 1. Signaling pathways in KEGG database used as basic networks

| Type | Name | |
| --- | --- | --- |
| Genetic Information Processing | Ribosome  RNA transport  Protein processing in endoplasmic reticulum  Spliceosome  Nucleotide excision repair  Base excision repair  DNA replication  Homologous recombination  Mismatch repair  RNA polymerase | SNARE interactions in vesicular transport  Protein export  Non-homologous end-joining  Sulfur relay system  Ubiquitin mediated proteolysis  mRNA surveillance pathway  Basal transcription factors  RNA degradation  Proteasome |
| Environmental Information Processing | ABC transporters  MAPK signaling pathway  ErbB signaling pathway  Calcium signaling pathway  Cytokine-cytokine receptor interaction  Neuroactive ligand-receptor interaction  mTOR signaling pathway  Wnt signaling pathway | Notch signaling pathway  Hedgehog signaling pathway  TGF-beta signaling pathway  VEGF signaling pathway  ECM-receptor interaction  Cell adhesion molecules (CAMs)  Jak-STAT signaling pathway |
| Cellular Processes | Cell cycle  Oocyte meiosis  p53 signaling pathway  Regulation of autophagy  Lysosome  Endocytosis  Phagosome | Peroxisome  Apoptosis  Focal adhesion  Adherens junction  Tight junction  Gap junction  Regulation of actin cytoskeleton |
| Immune System | Fc epsilon RI signaling pathway  B cell receptor signaling pathway  NOD-like receptor signaling pathway  RIG-I-like receptor signaling pathway  T cell receptor signaling pathway  Leukocyte transendothelial migration  Fc gamma R-mediated phagocytosis  Cytosolic DNA-sensing pathway | Natural killer cell mediated cytotoxicity  Antigen processing and presentation  Intestinal immune network for IgA production  Chemokine signaling pathway  Hematopoietic cell lineage  Complement and coagulation cascades  Toll-like receptor signaling pathway |
| Endocrine System | PPAR signaling pathway  Adipocytokine signaling pathway  Insulin signaling pathway  Melanogenesis | GnRH signaling pathway  Progesterone-mediated oocyte maturation  Renin-angiotensin system |
| Nervous System | Long-term potentiation  Neurotrophin signaling pathway | Long-term depression |
| Other Organismal System | Salivary secretion  Gastric acid secretion  Pancreatic secretion  Carbohydrate digestion and absorption  Protein digestion and absorption  Fat digestion and absorption  Bile secretion  Cardiac muscle contraction  Vascular smooth muscle contraction  Aldosterone-regulated sodium reabsorption | Vasopressin-regulated water reabsorption  Proximal tubule bicarbonate reclamation  Collecting duct acid secretion  Olfactory transduction  Taste transduction  Phototransduction  Dorso-ventral axis formation  Axon guidance  Osteoclast differentiation  Circadian rhythm - mammal |

Supplementary Table 2. Disease networks downloaded from KEGG database

| Type | Name |
| --- | --- |
| Cancers | Basal cell carcinoma  Endometrial cancer  Melanoma  Colorectal cancer  Chronic myeloid leukemia  Pancreatic cancer  Prostate cancer  Bladder cancer  Non-small cell lung cancer  Glioma  Renal cell carcinoma  Thyroid cancer  Small cell lung cancer  Acute myeloid leukemia |
| Immune Diseases | Asthma  Primary immunodeficiency  Systemic lupus erythematosus  Endometrial cancer  Autoimmune thyroid disease  Graft-versus-host disease |
| Neurodegenerative Diseases | Alzheimer’s disease  Parkinson’s disease  Huntington’s disease  Amyotrophic lateral sclerosis (ALS)  Prion diseases |
| Cardiovascular Diseases | Hypertrophic cardiomyopathy (HCM)  Dilated cardiomyopathy  Arrhythmogenic right ventricular cardiomyopathy (ARVC)  Viral myocarditis |
| Infectious Diseases | Hepatitis C  Shigellosis  Chagas disease (American trypanosomiasis)  Toxoplasmosis  Leishmaniasis  Malaria  Staphylococcus aureus infection  African trypanosomiasis  Epithelial cell signaling in Helicobacter pylori infection  Pathogenic Escherichia coli infection  Bacterial invasion of epithelial cells  Amoebiasis  Vibrio cholerae infection |
| Endocrine and Metabolic Diseases | Type I diabetes mellitus  Type II diabetes mellitus |

**Supplementary Table 3. The top 10 signaling pathways having close relationships with most diseases**

| Pathway name | Pathway category | Similarity score |
| --- | --- | --- |
| Intestinal immune network for IgA production | Immune system | 2.419 |
| T cell receptor signaling pathway | Immune system | 2.346 |
| Fc epsilon RI signaling pathway | Immune system | 2.016 |
| NOD-like receptor signaling pathway | Immune system | 1.920 |
| Toll-like receptor signaling pathway | Immune system | 1.675 |
| ErbB signaling pathway | Environmental information processing | 1.639 |
| B cell receptor signaling pathway | Immune system | 1.598 |
| Hematopoietic cell lineage | Immune system | 1.575 |
| Apoptosis | Cellular processes | 1.521 |
| Jak-STAT signaling pathway | Environmental information processing | 1.409 |

**Supplementary Table 4. The top 10 signaling pathways having poor relationships with most diseases**

| Pathway name | Pathway category | Similarity score |
| --- | --- | --- |
| Bile secretion | Digestive system | -2.609 |
| RNA degradation | Generic information processing | -2.467 |
| Peroxisome | Cellular processes | -2.345 |
| RNA transport | Generic information processing | -2.251 |
| Spliceosome | Generic information processing | -2.176 |
| mRNA surveillance pathway | Generic information processing | -2.050 |
| Lysosome | Cellular processes | -1.897 |
| Taste transduction | Sensory system | -1.884 |
| ABC transporters | Environmental information processing | -1.860 |
| PPAR signaling pathway | Endocrine system | -1.686 |


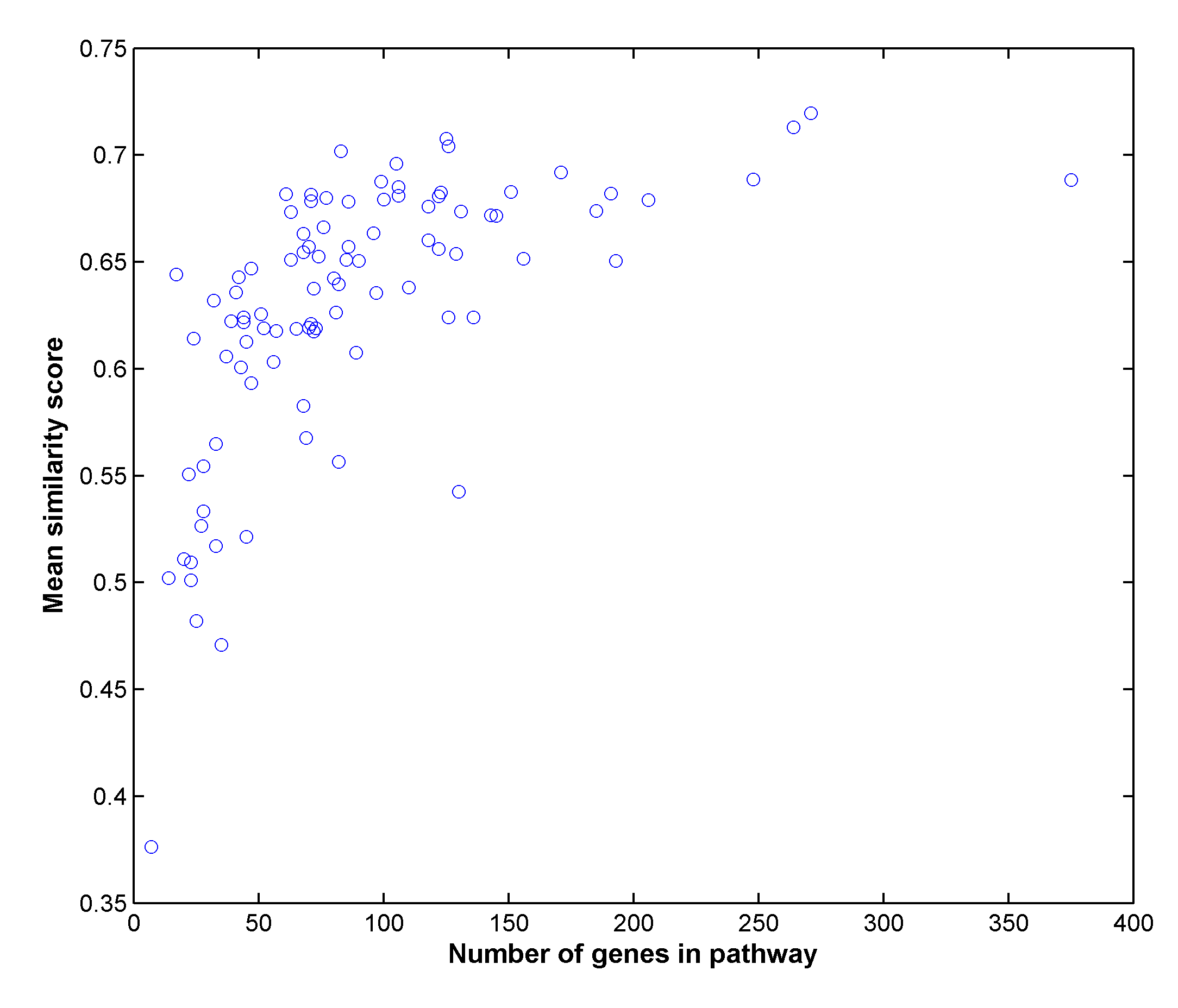


Supplementary Figure S1. The scatter graph of gene number in pathway and network similarity score.


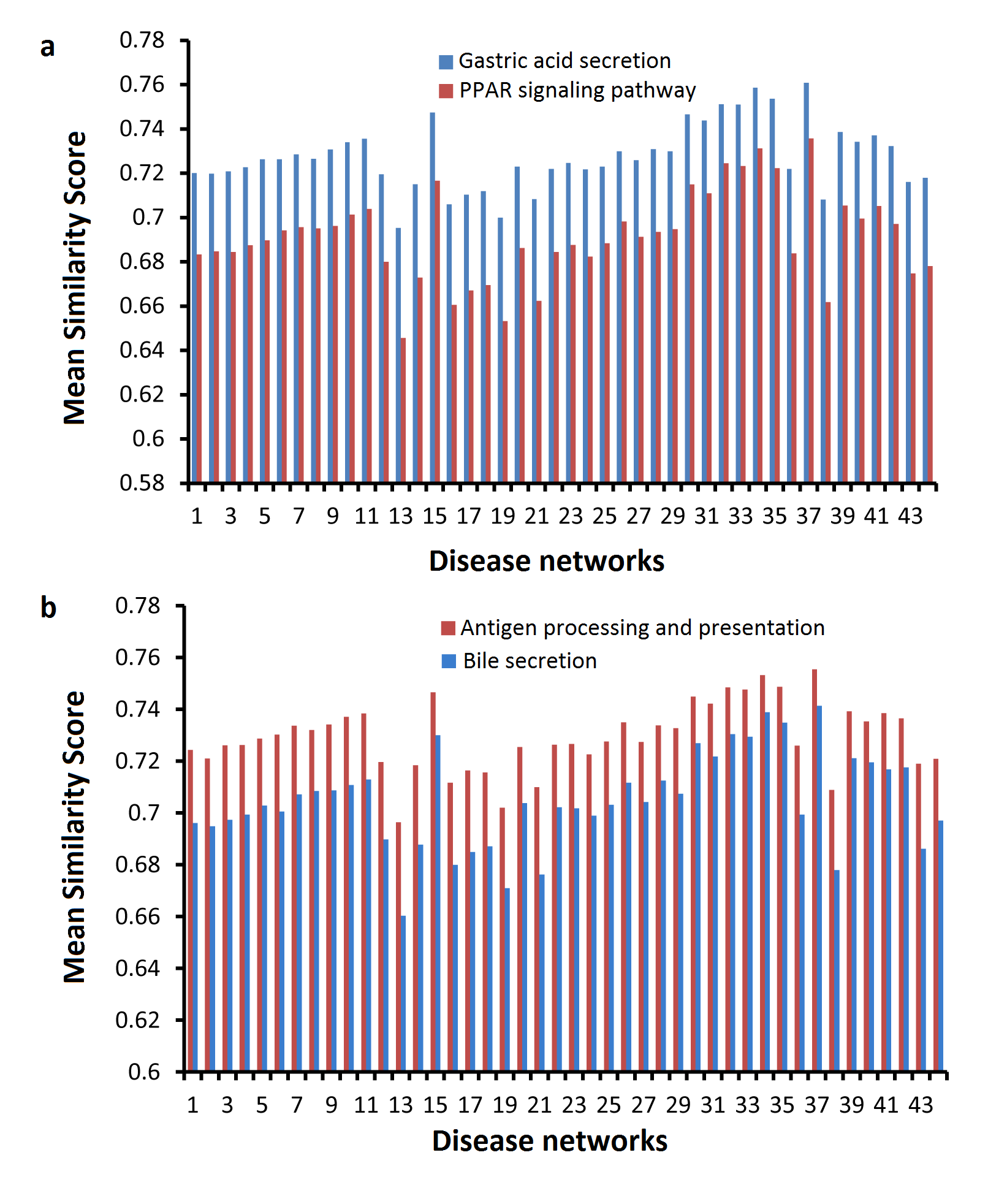


Supplementary Figure S2. The influence of network density on the similarity score. (a) The mean similarity score between random networks for two signaling pathways (Gastric acid secretion and PPAR signaling pathway) and the 43 disease networks. (b) The mean similarity score between random networks for two signaling pathways (Antigen processing and presentation and Bile secretion) and the 43 disease networks.
